# Application of semidefinite programming approach for detection of genomic copy number variations from peripheral blood in ovarian cancer

**DOI:** 10.3389/fonc.2026.1916039

**Published:** 2026-10-27

**Authors:** Michael Kalab, Arunabh Sharma, Anastasia Klopf, Lena Wahl, Huy Duc Le, Nina Hedemann, Annika Brauer, David Holthaus, Christoph Rogmans, Dirk Olaf Bauerschlag, Nicolai Maass, Marion van Mackelenbergh, Jörg Paul Weimer, Astrid Dempfle

**Affiliations:** 1 Institute of Medical Statistics, Kiel University and University Medical Center Schleswig-Holstein, Kiel, Germany; 2 Department of Gynecology and Obstetrics, Christian-Albrechts-University and University Medical Center Schleswig-Holstein, Kiel, Germany; 3 Department of Gynaecology, Jena University Hospital, Jena, Germany

**Keywords:** circulating tumor DNA (ctDNA), CNV, ddPCR, diagnostic, ovarian cancer, semidefinite programming

## Abstract

**Background:**

In 2025, we developed and published a detection test using digital droplet polymerase chain reaction (ddPCR) from cell-free DNA that indicates the presence of ovarian cancer (OC) in peripheral blood immediately before surgery with a sensitivity and specificity of 80%.

**Methods:**

By applying a new classification approach based on semidefinite programming (SDP), we were able to significantly improve the predictive power of this test, which consists of six ddPCR combinations that indicate genomic imbalances. In addition to the previously used ratio of the ddPCR combination products, we now also include the sum of the ddPCR combination products.

**Results:**

Data from the already published 57 controls without tumor background and a positive cohort of 15 OC were expanded by 5 additional OCs. With these data, we were able to define a separating boundary plane in a multidimensional space that better distinguishes the test results.

**Conclusions:**

This new method allows the detection of cell-free tumor DNA from OC with high tumor burdens in the plasma with high sensitivity and specificity. Further studies using larger and independent cohorts are needed to evaluate the robustness of our approach to evaluate its potential use as a screening algorithm and to assess the diagnostic potential of minimally invasive blood-based ddPCR tests in OC.

## 1 Introduction

The Center for Cancer Registry Data registered 6–709 new cases of ovarian cancer (OC) in Germany in 2022, which corresponds to an incidence of 9.9 per 100–000 women. About two-thirds of affected patients succumb to this disease, and only 44% survive the first five years (1). In 2022, 324–603 people worldwide were diagnosed with ovarian cancer, with an incidence of 6.7/100 000, and 206–956 died (2). Therefore, the treatment of OC is of particular importance in Germany. The high mortality rates are attributed to a very long, unnoticed and asymptomatic course of the disease. In most cases, the disease symptoms are only observed when the tumor burden is at an advanced stage which often results in a late diagnosis. However, early detection of OC leads to a better prognosis (3). Thus, a growing body of research has focused on developing methods that allow earlier diagnosis of the disease (4–6). These studies have primarily targeted genes that are known to have an influence on tumor development (7). Following the discovery of the clinical implications of mutated *BRCA* genes, the association between gynecological solid tumors and a deficient DNA repair mechanism soon became apparent (8, 9). This finding facilitated new approaches to treat OC (10), for example, using Poly(ADP-ribose)-Polymerase inhibitors (PARPi) (4, 11, 12). An intact DNA repair system requires a whole fleet of gene functions, where each gene influences the repair capacity of the DNA with varying weights. Ineffective DNA repair mechanisms lead to the accumulation of chromosomal translocations, deletions, and amplifications, ultimately resulting in CNVs and the loss of a purely diploid state. This creates genomic chaos within the cell, typically depriving it of the ability to survive in an organized manner. Hence, it is challenging to detect ovarian cancer using a single genetic marker due to the polygenic nature of the DNA repair capacity (13, 14). Many of these polygenic influences result in a common phenotype, characterized by a deviation of cells from the inconspicuous diploid state. This means that the more DNA repair genes fail, the greater the genomic chaos in the tumor cells. In 2014, Watkins et al. described this phenotype as genomic scarring as a consequence of an insufficient homologous DNA repair mechanism (4, 15). If demonstrated representatively, these genomic scars resulting from the development of genomic chaos could be suitable as markers for OC. According to the authors, the use of the resulting cellular phenotype, such as genomic chaos or the modification of the diploid character as a biomarker for OC is better suited instead of relying on individual gene markers. In this context, the evidence for the presence of OC can be provided by detecting circulating tumor DNA (ctDNA) in the patient’s peripheral blood system with typical copy number variations (CNV). In our previous study, we used this method to distinguish OC just prior to surgery from healthy controls, achieving a sensitivity and specificity of over 80% (16). To identify those genomic loci with the most and the fewest CNVs, we used array-based comparative genome hybridization (aCGH) on 30 OC patients in a first step published in 2025 (16). In a second step, we designed digital droplet PCR (ddPCR) assay combinations that amplify loci with many and few CNVs in OC respectively published in 2025 (16). Both ddPCR experiments in the linked test combinations were labeled with different fluorophores. In this ddPCR process, most of the up to 18 thousand droplets contain only one molecule as a template. As a result, CNVs lead to different numbers of differently colored ddPCR products in the droplets. If no CNVs are present as cell-free DNA in the blood, the number of differently labeled ddPCR products in the droplets should be approximately the same, which roughly represents a ratio of 1. Wahl et al. (2025) developed six ddPCR test combinations that significantly differentiated between the presence and absence of OC (16). A very simple procedure could show the presence of OC, in which at least 4 of the 6 test combinations showed significant differences in the number of ddPCR products published in 2025 (16). In further research approaches, we are trying to determine at what tumor burden this OC detection can be used for early detection or as a follow-up (17).

In each of the six test combinations published in 2025, ddPCR products from genomic regions with frequent CNVs and those with few CNVs in ovarian cancer are detected (16). Because the relationship between these two values becomes increasingly blurred as less cfDNA is available in the blood, we also included the sum of both values in the analysis to address this issue. This opens up the possibility of not only showing the ratio of ddPCR products in the result of a test combination, but also evaluating this result in relation to the total amount of cfDNA detected in this study for the first time. Thus, the ddPCR results of a test combination are entered into a two-dimensional coordinate system that maps ratios against quantities. This two-dimensional information is then combined in a multidimensional space encompassing all six test combinations. The new algorithm separates the data in this multidimensional space between ddPCR results from subjects without tumors and those from patients with ovarian cancer. In this study, we expanded the previously published cohort of 15 OC cases published in 2025 (16) by 5 additional cases, resulting in a sample size of 20, together with 57 healthy controls. Using the six ddPCR test combinations, we demonstrate the use of a quadratic classifier based on semidefinite programming (SDP) to distinguish patients and controls with improved sensitivity and specificity within this dataset.

## 2 Materials and methods

### 2.1 Cohort description

Our study comprised of a cohort of 20 patients of which 15 were previously published (16). Subjects were excluded from the study if they had a history of a tumor other than ovarian cancer or any wounds unrelated to ovarian cancer. To verify the fundamental detectability of OC via ddPCR, we are enrolling OC patients who are about to undergo surgery. Immediately before surgery, 8 mL of blood was collected from 20 patients with OC using a Paxgene Blood ccfDNA Tube (PreAnalytiX, Hilden, Germany, Cat. No. 768115). The blood samples stored at room temperature were used for cfDNA isolation within ten days to avoid cfDNA degradation. The cfDNA was isolated from the blood using a QIAamp DSP Circulating NA Kit (Qiagene, Hilden, Germany) according to the manufacturer’s instructions. Blood was also collected from 57 subjects without a tumor background using the same method. The biomaterial was used with the informed consent of the participants and with an ethics approval from the Medical Faculty of the Christian-Albrechts University of Kiel (D 420/23). The biosamples are part of the BMB-Gyn biobank, which is part of the PopGen 2.0 Network (P2N) biobank network in Kiel.

### 2.2 ddPCR tests

The basis for the selection of the test loci were the aCGH data from 20 OC cases, of which 15 have already been included in a previous publication (16). The ddPCR CNV tests were designed or selected from the website of BioRad (Hercules, CA, USA) and manufactured by BioRad. Fluorescin(FAM)-labeled ddPCR CNV assays of the genes *PAK2* in 3q29 (Assay ID: dHsaCNS290308003) and *PVT1* in 8q24.21 (Assay ID: dHsaCNS911399324) correspond to the loci with OC gains. FAM-labeled ddPCR CNV assays of the genes *Timm21* in 18q22.3 (Assay ID: dHsaCNS263027692), *MYOCD* in 17p12 (Assay ID: dHsaCNS286154155) and the region chr22q13.32 (Assay ID: dHsaCNS366711081) correspond to the genomic losses in OC. Hexachloro-Fluorescein(HEX)-labeled ddPCR CNV assays of the genes *JAK1* in 1p31.3 (Assay ID: dHsaCNS897218356) and *USP7* in 16p13.2 (Assay ID: dHsaCNS806739955) show rare alterations in OC. In the publication by Wahl et al. (2025) (16), all six of these test combinations clearly distinguish between OC patients and subjects with normal findings. These assessments are based on significant deviations and, specifically, values falling outside the 25^th^-75th percentile range of all measured fluorophore ratios from subjects without a tumor background. The test combinations used consist of *PAK2-JAK, PVT1-JAK, MYOCD-JAK, TIMM21-JAK, TIMM21-USP7*, and *CHR22-JAK*. Test combinations with patient cell-free DNA (cfDNA), with control DNA, and without templates were performed in technical triplicates. The test combinations were degenerated with HAEIII in a ddPCR assay for 15 minutes at room temperature. The samples were then separated into 10–000 to 18–000 individual droplets in the droplet generator (BioRad, Hercules, CA, USA) using appropriate oil. The cfDNA amplification was performed in a 96-well plate in a C1000 Touch Thermal Cycler (BioRad, Hercules, CA, USA). The enzymes of the master mix were activated for 10 minutes at 95 °C. The template DNA was denatured at 94 °C for 30 s. The amplification of the single-stranded cfDNA was carried out at increasing temperature at 2 °C/s starting at 60 °C for one minute. Denaturation and amplification were repeated 49 times. Finally, the polymerase was inactivated for 10 minutes at 98 °C and left at 4 °C for at least 30 minutes. Droplets stored at 4 °C were usually measured the next day.

### 2.3 ddPCR-analysis

The signal color and the number of droplets were measured using a QX200 Droplet Reader (BioRad, Hercules, CA, USA). The data and the concentrations of the color-positive droplets were evaluated and calculated using QuantaSoft Ver. 1.2 software (BioRad, Hercules, CA, USA). Because ddPCR can detect individual molecules within the droplets, which then serve as templates for a PCR reaction, there are no threshold values as in qPCR. Each positive signal represents a single molecule serving as a template for the PCR reaction and is counted.

### 2.4 Statistical analysis

To evaluate the blood samples, we calculated the mean number of droplets with a positive signal based on technical triplicates for all assays and both FAM and HEX channels. In total, 10 values were missing, which were imputed using the mean of the respective groups (OC cases and controls). We applied a natural logarithm (log) transformation to normalize the distributions of the concentration values of FAM and HEX. Next, we calculated the ratio of the log(FAM) to log(HEX) means (FAM/HEX) and observed that the patients with a high total number of positive droplets showed less pronounced ratios. So, as a next step, we also considered the sum of the log(FAM) and log(HEX) means (FAM+HEX) for each assay, what is new compared to the 2025 publication (16).

Unlike in previous publications, we used a quadratic classifier based on semidefinite programming (SDP) to obtain an optimal separating hyperplane to distinguish OC patients and controls based on data from the FAM and HEX assays. This idea of class separation is based on the use of support vector machines (SVMs) developed by Cortes and Vapnik (18) for binary classification tasks. SVMs have a clear geometric interpretation: they determine an optimal separating hyperplane that minimizes penalties for misclassified data points, which are typically measured as the distance from the hyperplane (19). One can optimize the performance of the SVM by applying an appropriate kernel function, which embeds data into a higher-dimensional space. Choosing a suitable kernel is crucial, and kernel-based methods generally perform well due to their ability to incorporate prior knowledge.

Our main idea was to enclose case samples within a convex set while keeping control samples outside. In the case of a second-order polynomial kernel, it would be required for the coefficient matrix of the quadratic terms to be positive semidefinite. Accordingly, for our study, we formulated and solved a semidefinite program (SDP) to obtain the separating hyperplane. Since false negatives are more critical than false positives in a screening context, we introduced a hyperparameter μ, with 0 < μ < 1, to allow adjustment for either high sensitivity (μ close to 1) or high specificity (μ closer to 0) in cases where perfect separation was not possible. We represent cases and controls as vectors in R^n^, denoting the set of cases by *S* and the set of controls by *H*, and let ϵ > 0 (its exact value is not crucial). The SDP is formulated as:


$$\begin{array}{cc} \text{(SDP) min} & \mu \sum_{s\in S}z_{s}+\left(1-\mu \right)\sum_{h\in H}z_{h}\\ \text{subject to} & s^{T}Xs+s^{T}x+x_{0}-z_{s}\leq -\epsilon \text{ for all }s\in S\text{ (R1)}\\ h^{T}Xh+h^{T}x+x_{0}+z_{h}\geq \epsilon \text{ for all }h\in H\text{ (R2)}\\ z\geq 0,X\;\in R^{n\times n\;}\text{positive semidefinite,}\\ x\in R^{n},\;x_{0}\in R,\;z\in R^{|S|+|H|.} \end{array}$$

Since (SDP) is a convex optimization problem, any solution is guaranteed to be a global minimum.

Let $\left(X_{\mathrm{opt}},x_{\mathrm{opt}},x_{0,\mathrm{opt}}\right)$ be a minimizer of (SDP).


$$s^{T}X_{\mathrm{opt}}s+s^{T}x_{\mathrm{opt}}+x_{0,\mathrm{opt}}=0$$

can be rewritten as an ellipsoid: ${\left(s-s_{0}\right)}^{T}A\left(s-s_{0}\right)=1$, where


$$s_{0}=-\frac{1}{2}X_{\mathrm{opt}}^{-1}x_{\mathrm{opt}}\text{ and }A=X_{\mathrm{opt}}/\left(s_{0}^{T}X_{\mathrm{opt}}s_{0}-x_{0,\mathrm{opt}}\right)$$

Solutions of this form are shown in the results section.

## 3 Results

Our study used six ddPCR assays to quantify cell-free DNA from 20 OC patients and 57 healthy controls to detect somatic CNVs in OC using a multidimensional ellipsoid classification algorithm.

### 3.1 Correlation between assays

We used two features for each combination: the ratio of log(FAM) to log(HEX), which captures relative CNV gain or loss, and the sum of log(FAM) and log(HEX), which captures absolute cfDNA abundance. We first looked at the correlation structure for the ratios and the sums of the log-transformed concentration values of the fluorophores FAM and HEX in all possible combinations. Among the ratios, the correlation between PVT1/JAK and MYOCD/JAK was the highest (Spearman’s ρ = 0.906). Nine of the fifteen pairwise correlations exceeded 0.8, while six fell below this threshold: (i) the five pairs involving TIMM21/JAK (ρ = 0.727-0.786) and (ii) the pair TIMM21/USP7-PAK2/JAK (ρ = 0.778). Overall, the ratio-ratio correlations ranged from 0.727 to 0.906. This indicates that the ratios carry partly overlapping but not fully redundant information across combinations, (**Figure 1**). For several combinations, the pairwise ratio correlations were higher in patients than in the controls. This was most evident for pairs involving the TIMM21 ratios, as exemplified by TIMM21/USP-PAK2/JAK (ρ = 0.931 in patients vs. 0.687 in controls), TIMM21/JAK-TIMM21/USP7 (0.878 vs. 0.705) and TIMM21/JAK-MYOCD/JAK (0.811 vs. 0.669), while other pairs were comparable between the two groups, as for MYOCD/JAK-CHR22/JAK (0.872 vs. 0.871). This pattern is consistent with genomic instability affecting multiple loci simultaneously. It must be noted, however, that the overall correlations remained high in both groups and that the sample is small. We present the observed differences as an exploratory observation rather than a formal effect. Similarly, the sums of the FAM and HEX values were highly correlated with an overall correlation above 0.9 for all combinations. However, the sums and the ratios showed low negative correlation between them. Among them, TIMM21+JAK and TIMM21+USP7 were most correlated (r = -0.698) while others ranged between -0.4 to -0.6. These correlations, stratified by patients and controls groups are shown in **Supplementary Figure 1**.

**Figure 1:**
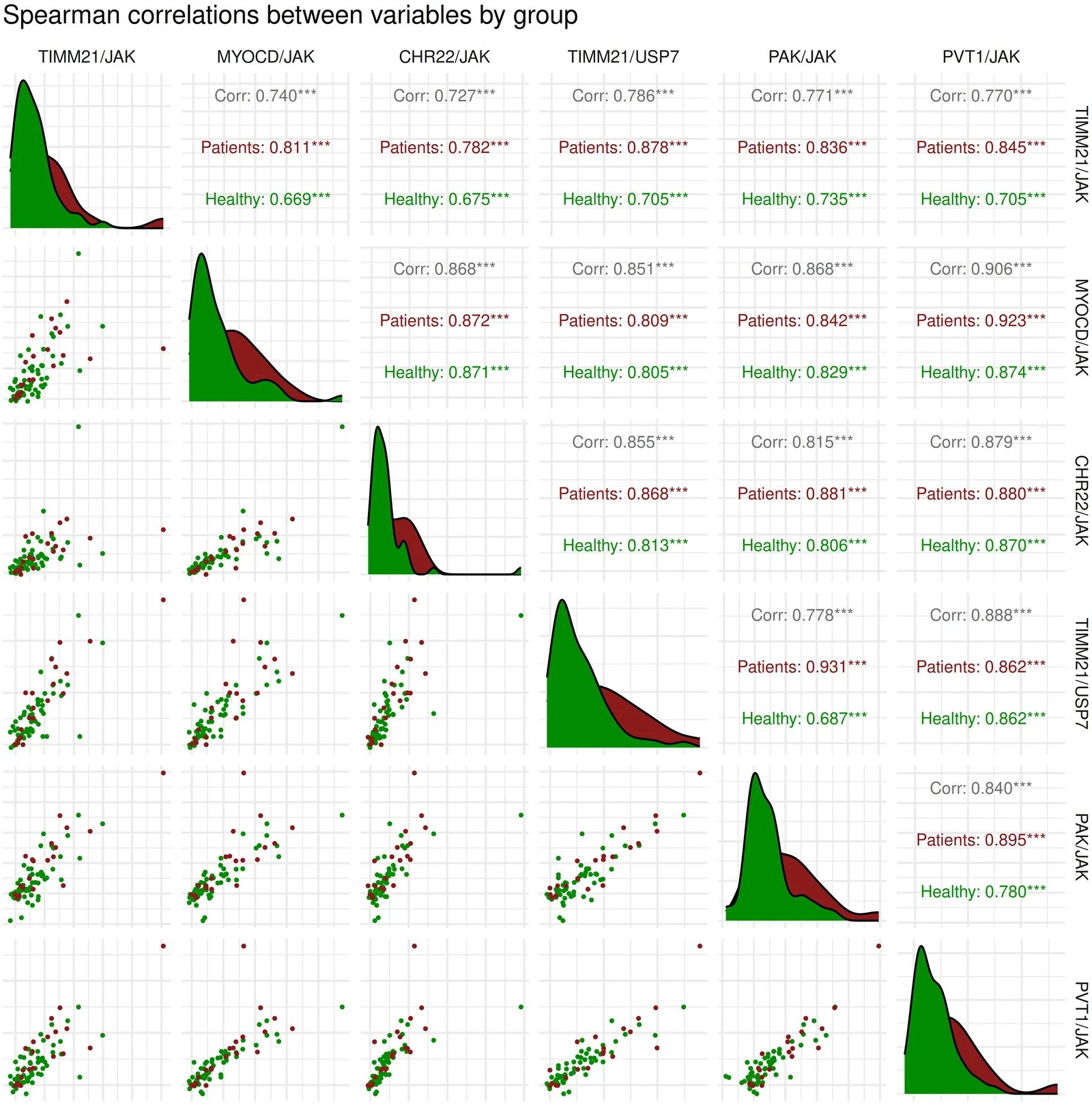
Pairwise Spearman correlation matrix and scatter plots for log(FAM) and log(HEX) assay ratios among OC patients (dark red) and healthy controls (green).

For our analysis, we used both the ratios and the sums of the log-transformed FAM and HEX assays. Since sums capture absolute abundance shifts and effects of overall cfDNA concentration, and ratios capture relative CNV gain or loss, it was useful to include both these metrics as it allowed the algorithm to detect subtle CNV patterns which can occur in cell-free DNA. The SDP-based ellipsoid classifier and the sensitivity-tuning hyperparameter μ are described in the Statistical analysis section.

### 3.2 Diagnostic performance across assays

As a preliminary step, we evaluated each FAM/HEX combination separately. For this, the ratios and the sums of the log-transformed concentration values of the fluorophores FAM and HEX for each assay were used as primary descriptors, as they underscore the discriminative feature of CNV signals relative to reference DNA. We then applied the ellipsoid algorithm having a classification boundary to minimize false negatives. We fitted the ellipsoidal boundary to enclose nearly all the OC cases. The small number of controls that fell within the boundary were counted as false positives. To quantify the ability of each assay to discriminate OC patients from healthy controls, we used the receiver operating characteristic (ROC) analysis, applied over a range of the sensitivity-tuning hyperparameter μ. Among test combinations, PVT1-JAK achieved the highest diagnostic accuracy (AUC = 0.952), followed closely by TIMM21-USP7 (AUC = 0.949), PAK2-JAK (AUC = 0.932), and CHR22-JAK (AUC = 0.931). MYOCD-JAK and TIMM21-JAK yielded lower AUCs of 0.899 and 0.785, respectively (**Figure 2**). From this analysis, we chose an optimal value of μ that maximizes sensitivity while maintaining a relatively high specificity. For these values of μ, we created two-dimensional feature plots wherein we project the OC patients and controls together, with the ellipsoid algorithm having a classification boundary to minimize false negatives. **Figure 3** shows the two-dimensional ellipsoid boundaries for PVT1-JAK (μ =0.868) and PAK2-JAK (μ = 0.888), both achieving a sensitivity of 0.95 each and specificities of 0.77 and 0.63, respectively.

**Figure 2:**
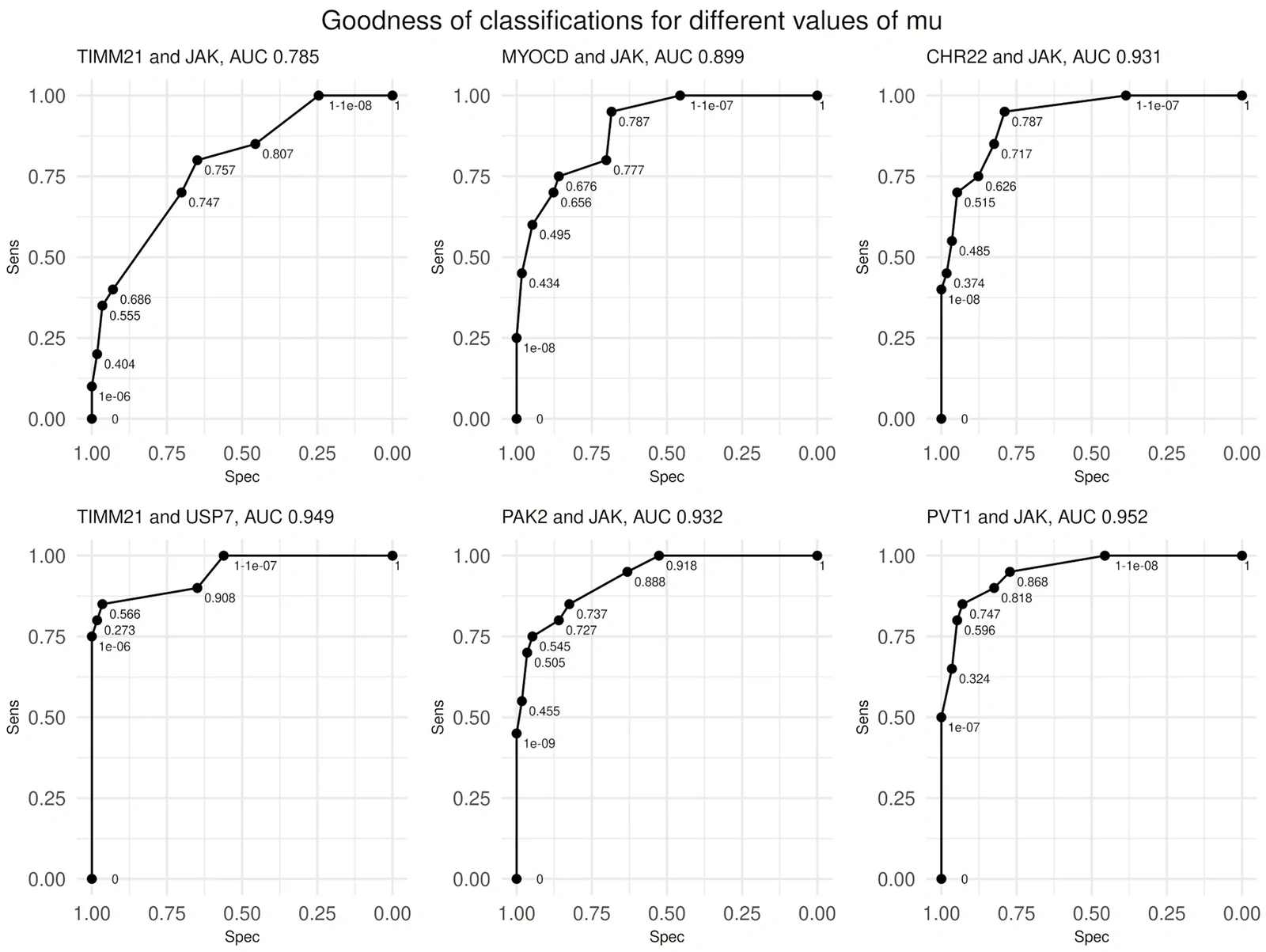
ROC curves depicting sensitivity and specificity along with AUC values for individual assays across hyperparameter values (μ).

**Figure 3:**
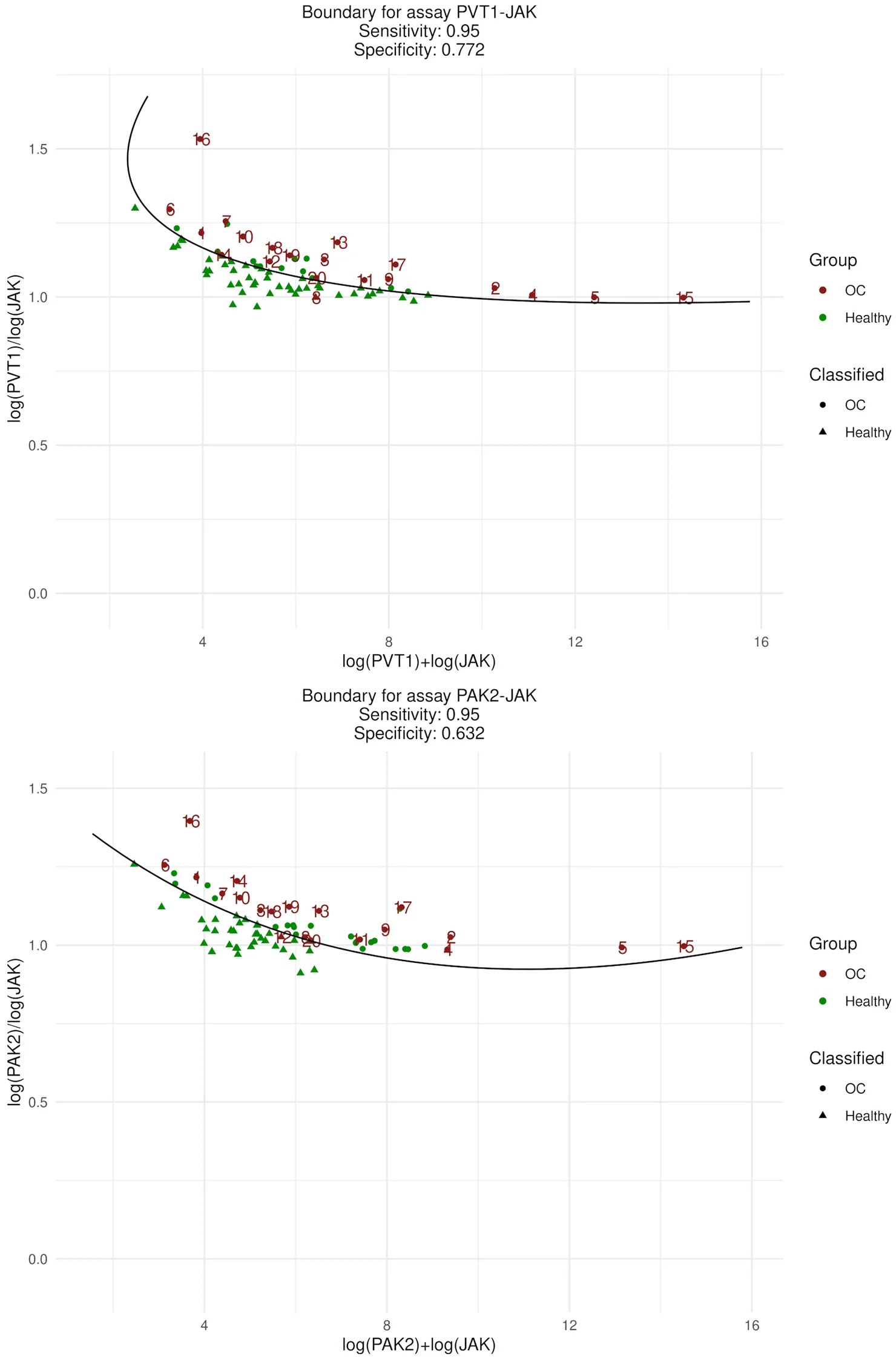
Two-dimensional ellipsoid boundaries for the test combinations PVT1-JAK and PAK2-JAK. For each combination, the sum of the log-transformed concentrations of the two assays is plotted on the x-axis, and their ratio on the y-axis. Each point represents one participant (OC in dark red, controls in green), while the curve is the fitted ellipsoidal boundary. PVT1-JAK achieved a sensitivity of 0.95 and a specificity of 0.772 (μ = 0.868), while PAK2-JAK achieved a sensitivity of 0.95 and a specificity of 0.632 (μ = 0.888).

Discrimination improved markedly when combining two or more assays. By combining four or more assays in the ellipsoid algorithm, we could accomplish a perfect separation of OC patients from control samples in this cohort. The optimal assay combinations (**Figure 4**) show several distinct sets of four assays which provide both 100% sensitivity and 100% specificity. Seven distinct four-assay combinations achieved perfect separation in this cohort: (i) TIMM21-JAK, MYOCD-JAK, CHR22-JAK and TIMM21-USP7; (ii) TIMM21-JAK, MYOCD-JAK, CHR22-JAK and PAK2-JAK; (iii) TIMM21-JAK, MYOCD-JAK, CHR22-JAK and PVT1-JAK; (iv) TIMM21-JAK, MYOCD-JAK, TIMM21-USP7 and PAK2-JAK; (v) TIMM21-JAK, MYOCD-JAK, PAK2-JAK and PVT1-JAK; (vi) MYOCD-JAK, CHR22-JAK, TIMM21-USP7 and PAK2-JAK; and (vii) MYOCD-JAK, CHR22-JAK, TIMM21-USP7 and PVT1-JAK. MYOCD-JAK was present in all seven combinations.

**Figure 4:**
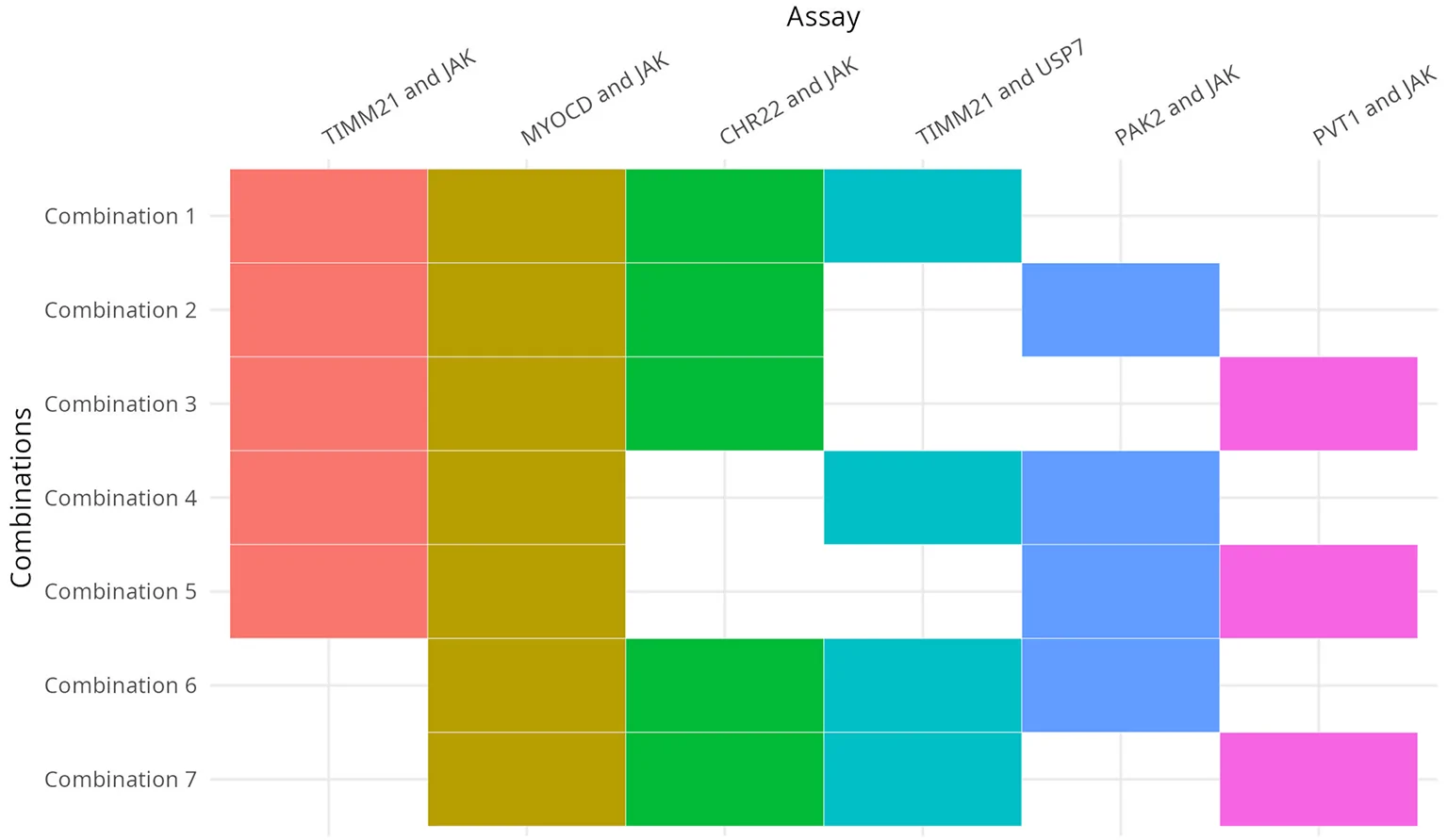
Heat map of all four-assay combinations allowing perfect case-control separation in high-dimensional ellipsoid space.

## 4 Discussion

There is an unmet need for improved treatment options in ovarian cancer, which can be achieved through early detection of the disease (17, 20). This requires a way to clearly identify the tumor. The detection of OC presented here is reliably possible in patients with a high tumor burden immediately before surgery. However, to determine whether the test is also suitable for early detection, it needs to be tested on patients with a lower or very low tumor burden in early stages. Since all patients analyzed here presented with a high tumor burden, our results pertain to detectability where ctDNA is most abundant and do not establish performance in early-stage disease. Given this background, our current approach may be more directly applicable to disease monitoring and recurrence detection, where a measurable tumor mass is expected. Hence, any future assessment studies of early detection will therefore require stratification of patients by tumor stage. Only if the tumor can be clearly identified in the blood does this tool offer the possibility of detecting early stages of the tumor in a blood sample (21). The cancer antigen CA125 is used to help confirm a suspected OC (22). However, an increase in this glycoprotein can also be detected in blood plasma during pregnancy, in benign diseases and during menopause (22–24). Therefore, this test for the detection of OC achieves a sensitivity of only 63-95% and a specificity of 82-95%. Unlike this glycoprotein, the detection of somatically occurring genomic imbalances has a more stringent potential to indicate tumors. This stringency exists because deviations from diploid or haploid autosomal equilibrium in human cells outside the germline have so far only been detected in tumor cells (25–28). In this exploratory study, we show that it is possible to detect ovarian cancer with very high reliability using a combination of our ddPCR tests. Past studies have attempted to detect specific gene mutations of a tumor entity (29–31). Our approach, however, is to detect genomic imbalances in the tumor and thus a phenotype of the rearranged OC cells (32, 33). Here, applying a classification algorithm based on semidefinite programming, we identified seven distinct groups of four ddPCR assay combinations that could correctly classify between OC patients and controls. We chose a classifier based on semidefinite programming for three reasons. First, the problem is convex, so any solution is a global optimum and does not depend on initialization. Second, the positive semidefinite constraint yields a closed, convex ellipsoidal boundary, which suits our data: control samples resemble normal diploid DNA, while tumor samples deviate from it and can be enclosed within the ellipsoid. Third, the parameter μ can be tuned to balance the sensitivity-specificity trade-off, which is desirable in screening, where false negatives are more costly than false positives.

In addition to the improvement of this analysis algorithm, the ability to detect the tumor-typical loss of diploidy in ovarian tumor cells using ddPCR represents a highly significant finding of this study. Genetic alterations particularly accumulate in solid tumors (34–36) and have varying degrees of influence on the development of the tumor. Genetic mutations are specifically searched for in various approaches for personalized medicine (37, 38). For example, mutations in the BRCA1/2 genes in ovarian tumor tissue open up the possibility of treatment with PARP inhibitors. However, such BRCA1/2 mutations cause only a small percentage of deficiencies in the homologous DNA repair system (HRD) (36). Therefore, other genes that are also involved in the DNA repair system are being investigated. For a definite diagnosis of HRD, its phenotype is often determined using aCGH (Myriad myChoice^®^ HRD Test) in addition to sequencing (37–39). Additionally, individual, typical gene mutations are not ideal markers for tumor detection (35, 36, 38). In solid tumors, the accumulated gene mutations are simply too heterogeneous to achieve sufficient relevance (40). To detect genetic tumors, it is not suitable to find an individual, unrepresentative gene mutation. Instead, the ideal approach would be to find the genetic marker that is present in all solid tumors and is distinct from the healthy tissue: the genomic imbalance, which no longer has a diploid character (37, 41, 42). During apoptotic, senescent and macerating processes, a cell releases parts of its genomic DNA into the extracellular space and thus ultimately into the peripheral blood system (29, 40, 43, 44). Imbalances in this released DNA should be reflected, depending on its degree of dilution, against the background of cell-free DNA derived from diploid cells. The DNA released by such dying cells, with its genomic imbalances, would be detectable to varying degrees of dilution level against the background of cell-free DNA from diploid cells (16). An important aspect of our study is the use of two features derived from the (log-transformed) ddPCR data for each test combination: the ratio of positive droplets in FAM to those in the HEX assay, and the sum of positive droplets from both assays. The ratio captures the relative copy number variations, and the sum tells us the absolute abundance of target DNA molecules in the sample. By employing both features, we improve the classification stability and accuracy, especially where the DNA concentrations are low and the ratio alone could become noisy due to stochastic variation in droplet counts.

We would like to emphasize that our analysis is exploratory and based on data from a relatively small number of OC patients, which is a key limitation. This may lead to overfitting of the algorithm and thereby overestimate the classification performance. Because the classifier was both developed and evaluated within the same cohort, without an independent validation set, the reported sensitivity and specificity should be interpreted with caution. These estimates are best regarded as hypothesis-generating until they are confirmed in larger, independent cohorts. The limited number of cases likewise means that the study is not statistically powered for definitive performance estimates. Nonetheless, we introduce, for the first time, a classification approach based on semi-definite programming in the detection and screening of OC patients. Here, we have also incorporated an adjustable parameter (μ) that can be utilized to prioritize sensitivity, which can be useful in initial screening and classification of OC patients. Future studies using larger and independent cohorts will be useful in assessing the robustness of our approach and evaluating its potential use as a screening algorithm in clinical settings.

The detection of OC at earlier stages and understanding disease progression through blood-based testing are clinically important as both are associated with better treatment outcomes. To address this, current projects are underway to obtain plasma samples collected from patients during the course of the treatment, with particular focus on time points when disease recurrence leads to an increase in tumor mass. Additionally, medical imaging measurements of the increase in tumor mass can be compared with variations in droplet concentrations detected by ddPCR assay. Moreover, the sensitivity of ddPCR tests relative to tumor mass can be evaluated by examining ovarian cysts, of which some may develop into malignant tumors. These malignant growths could allow us to understand the correlation between tumor mass and ddPCR detectability in OC (42). Ongoing studies are underway to understand the diagnostic potential of minimally invasive blood-based ddPCR tests in OC.

## 5 Conclusions

The new algorithm presented here differentiates between patients with OC and subjects without a tumor background with improved, high reliability. In particular, considering both the ratio of CNV-rich to CNV-poor regions of the OC and their sum allows for a clear separation in the multidimensional data space of six test combinations. Within this cohort, the method separated OC with a high tumor burden from tumor-free controls with high sensitivity and specificity. If it becomes possible to determine the detection limit at lower tumor burdens, this method could be used to detect OC earlier.

## Data Availability

The bio samples are the property of the BMB-GYN bio sample bank, which is part of the p2n biobank network in Kiel and was funded by the German Federal Ministry of Education and Research (BMBF grant 01EY1103). The datasets used and/or analysed during the current study are available from the corresponding author on reasonable request.

## Supplementary material

Supplementary Figure 1 Spearman correlation between the ratios and the sums of the log-transformed concentration values of the fluorophores FAM and HEX in all possible combinations.
