# Supplementary material for: Application of semidefinite programming approach for detection of genomic copy number variations from peripheral blood in ovarian cancer
Source: Front Oncol. 2026 Oct 27;16:1916039. doi: 10.3389/fonc.2026.1916039 (PMC13724512; doi:10.3389/fonc.2026.1916039)

Spearman correlations between variables by group

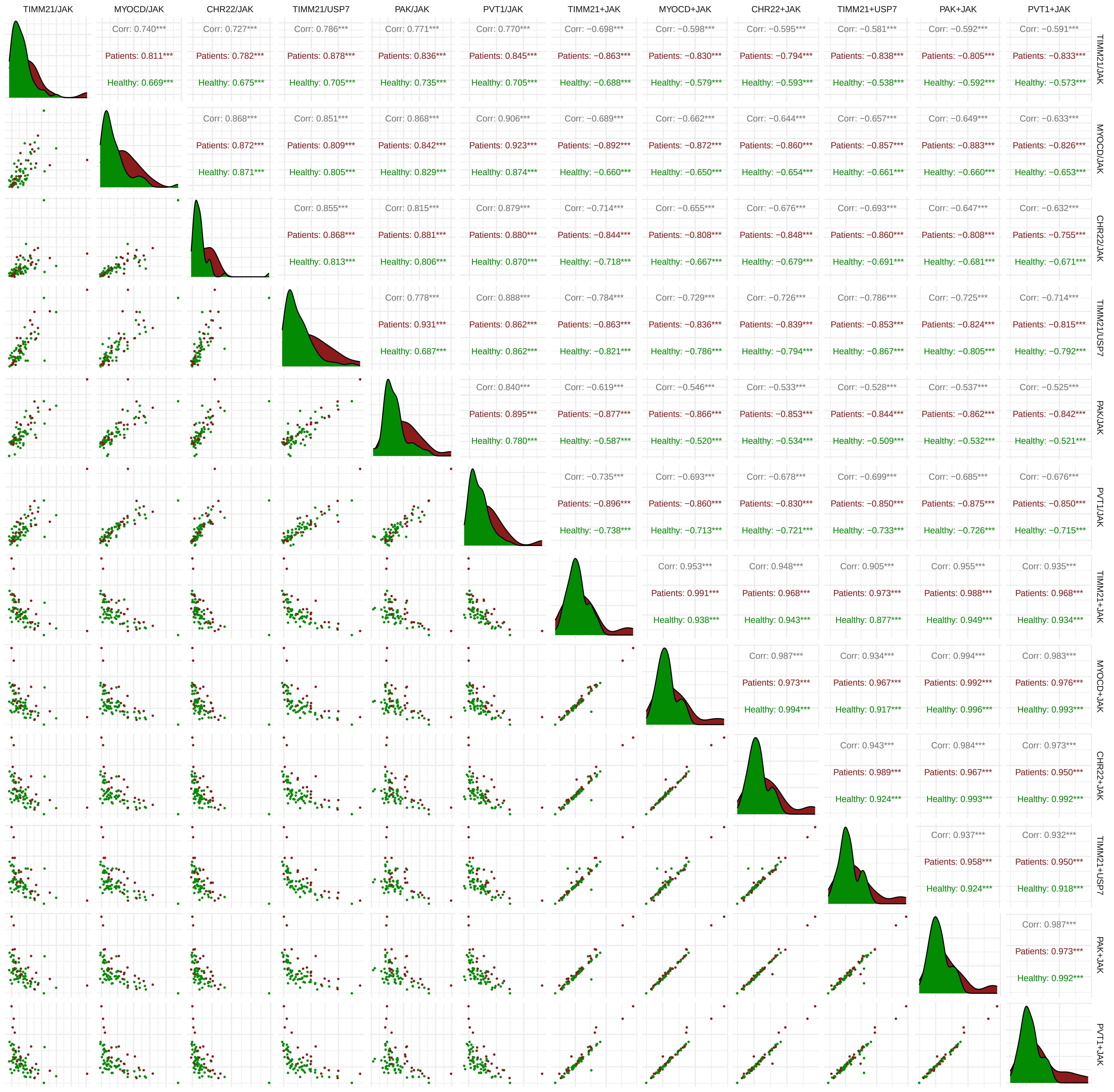

Supplement: Supplementary file 1 [file DataSheet1.pdf]
